# Supplementary material for: How Should Job Crafting Interventions Be Implemented to Make Their Effects Last? Protocol for a Group Concept Mapping Study
Source: Int J Environ Res Public Health. 2022 Oct 26;19(21):13922. doi: 10.3390/ijerph192113922 (PMC9654425; doi:10.3390/ijerph192113922)
Supplement: Supplementary file 1 [file ijerph-19-13922-s001.zip › ijerph-1941441-supplementary.pdf]

Supplementary file 1 Reporting guidelines

Good Reporting of A Mixed Methods Study (GRAMMS; O'Cathain et al., 2008)

Item	Page Reported
(1) Describe the justification for using a mixed methods approach to the research question	4
(2) Describe the design in terms of the purpose, priority and sequence of methods	4-7
(3) Describe each method in terms of sampling, data collection and analysis	4-7
(4) Describe where integration has occurred, how it has occurred and who has participated in it	N/A – concept mapping is a self-contained mixed method
(5) Describe any limitation of one method associated with the presence of the other method	N/A – concept mapping is a self-contained mixed method
(6) Describe any insights gained from mixing or integrating methods	N/A – study protocol

Concept Systems guidelines for reporting concept mapping (Concept Systems, Inc.)

Because this is a study protocol, below we indicate where plans for the recruitment, procedure, and analysis are described:

Item	Page Reported
A. Essential components of describing GCM data collection	
(1) Description of your participants (who did you invite, who participated and at which phases of the project)	4
(2) The focus prompt for the project	5
(3) Description of how you collected the ideas	5
(4) Brief explanation of idea synthesis and the final statement set	5-7
(5) Description of how participants sorted (online or in-person)	5
(6) The rating scales posed to participants	5
B. Essential components of describing GCM Results	
(1) The stress value computed for the multi-dimensional scaling representation of the sort data	6
(2) The final number of clusters chosen	N/A – study protocol
(3) The story the map told you	N/A – study protocol
(4) Discussion of how the ratings overlay onto the conceptual framework.	N/A – study protocol

References

O'Cathain A, Murphy E, Nicholl J. The quality of mixed methods studies in health services research. J Health Serv Res Policy. 2008;doi:10.1258/jhsrp.2007.007074

Concept Systems, Inc. Group concept mapping resource guide. 2015. https://groupwisdom.com/gcmrg#:~:text=and%20software%20guides.-,Dissemination,-You%20have%20completed. Access September 2022.
